# Health Tract, No. 216

**Published:** 1864-09

**Authors:** 


### FETID FEET.

Some persons can be "smelled" a mile off, more or less; it is a misfortune, and a source of very great mortification to the refined and sensitive. It may be "born" with some; with others, if not all, it is the result of a diseased condition of the system, or of a neglect of personal cleanliness. There is a peculiar odor emanating from the feet which is, perhaps, always the result of uncleanliness. If daily washings do not remove these odors, a very efficient wash is found in red oxide of lead, one part, to twenty-nine parts of the liquor of the sub-acetate of lead; the first to be bruised in a porcelain mortar, gradually adding the latter; apply a few drops once a week, oftener in summer.

A specific odor escapes every one, and is peculiar to the individual; the dog knows it, and by it follows his master through any crowd of human beings, and never makes a mistake. A man's organ of smell is not thus acutely developed; still there are persons whose peculiar penetrating odor is readily recognized. This does not come from the "sweat" of the person, as no such odor issues from the hands, but from the arm-pits and other parts kept covered by the clothing, so that the air can not penetrate; nor is the application of soap and water too frequently allowed. When the "sweat" remains in contact with the skin, it undergoes a chemical change, and it is this which disengages the peculiarly disagreeable odor, as to the feet particularly; thus this chemical formation is a kind of fetid fat, which is absorbed into the pores of the leather, and there it is detained with fresh additions daily, for weeks and months, with increasing rancidity, as the smell of any old boot or shoe will demonstrate. Some persons wear stockings without change from the time they are first put on until they are worn full of holes. Very many do not wash their feet oftener than once a month; only a few as often as once a week. The feet ought to be washed every night before going to bed, and no stocking, boot, or shoe should be put on a second time, until it has had a whole day's sunning, at least by those who have an ambition to be and feel as sweet and clean as a dew-drop on the rose of summer, or put two tablespoons of the compound spirits of ammonia (hartshorn) in a basin of water, and wash the face, hands, arms, armpits, and feet with it. The skin is left fresh, clean, and sweet; it is perfectly harmless, and costs but little.

